# The exposure to volatile organic compounds associate positively with overactive bladder risk in U.S. adults: a cross-sectional study of 2007–2020 NHANES

**DOI:** 10.3389/fpubh.2024.1374959

**Published:** 2024-06-07

**Authors:** Dawen Zhang, Ziyi Yan, Junhao He, Yunmin Yao, Kai Liu

**Affiliations:** Department of Urology, The Fifth People's Hospital of Wujiang District, Suzhou, China

**Keywords:** overactive bladder, volatile organic compounds (VOCs), cross-sectional studies, environmental pollution, National Health and Nutrition Examination Survey

## Abstract

**Objective:**

The aim of this study was to comprehensively investigate the potential relationship between blood volatile organic compounds (VOCs) and overactive bladder (OAB) risk.

**Methods:**

A total of 11,183 participants from the 2007–2020 National Health and Nutrition Examination Survey (NHANES) were included in this cross-sectional study. We used multivariate logistic regression models to investigate the relationship between nine blood VOCs and OAB risk. Restricted cubic spline (RCS) analysis was used to investigate the dose-response relationship between blood VOCs and OAB. In addition, the overall association of blood VOCs with OAB risk was assessed by weighted quantile sum (WQS) regression model. Finally, we conducted subgroup analyses to explore the findings in different high-risk populations.

**Results:**

After adjusting for potential confounders, logistic regression analysis revealed that blood 2,5-dimethylfuran (aOR = 2.940, 95% CI: 1.096–7.890, *P* = 0.032), benzene (aOR = 1.460, 95% CI: 1.044–2.043, *P* = 0.027) and furan (aOR = 9.426, 95% CI: 1.421–62.500, *P* = 0.020) were positively independent associated with the risk of OAB. And dose-response risk curves indicated that 2,5-dimethylfuran, benzene and furan in the blood were linearly positive associated with OAB risk. WQS regression analysis showed that exposure to mixed blood VOCs increased the risk of OAB (OR = 1.29, 95% CI: 1.11–1.49), with furans having the greatest weight. In subgroup analyses, we found that OAB was more susceptible to blood VOCs in young and middle-aged, male, non-hypertensive, and alcohol-drinking populations.

**Conclusions:**

The results of this study indicate that high exposure to VOCs is independently and positively associated with OAB risk in U.S. adults, particularly 2,5-dimethylfuran, benzene, and furan. In addition, age, gender, hypertension and alcohol consumption may influence the association. Our study provided novel epidemiologic evidence to explore the potential role of environmental pollutants in OAB.

## Introduction

Volatile organic compounds (VOCs) consist of a group of tiny organic molecules in different forms and are widely found in human daily life (1). VOCs can be produced not only naturally, but also through many human activities, including plants, animals, fuel combustion, production of paints, solvents, and more (2, 3). Widespread airborne VOCs result in easier and more common exposures to the general population than other pollutants to which people are exposed in specific environments (4, 5). Due to their characteristics of volatilization, solubility in water and adherence to particles, VOCs can be unintentionally absorbed through inhalation, dermal contact and ingestion through contaminated water and food (6, 7). Therefore, individual VOC exposure levels can be assessed by measuring blood and urine levels of VOCs and their metabolites (8). A growing body of research suggests that exposure to VOCs is associated with a variety of health disorders, including respiratory, neurological disorders, and diabetes (3, 9, 10). In fact, VOCs can not only be directly absorbed by the human body, resulting in health hazards, but can also be converted into high-activity volatile substances through chemical reactions, facilitating the formation of photochemical smog, exacerbating environmental pollution, and further harming human health (10, 11). In urologic diseases, the impact of VOCs on the disease progression has also received increasing attention from researchers. Early detection of exposure to VOCs has been found to aid in the diagnosis and detection of bladder cancer (12). In addition, the measurement of VOCs in urine was found to be helpful in the diagnosis of kidney cancer in a single-center study and is a promising clinical diagnostic modality (13).

In 2002, the International Continence Society (ICS) defined overactive bladder (OAB) as a storage symptom syndrome which is characterized by “urgency, with or without urgency urinary incontinence (UUI), usually accompanied by increased daytime urinary frequency and nocturia” (14). According to the NOBLE program in the United States, the prevalence of adult OAB is 16% for men and 16.9% for women (15). In Europe, epidemiologic statistics show that the OAB prevalence among adults over 40 years of age is 16.6% (16). OAB, as a global epidemic of chronic functional bladder disorders, significantly affects the physical health and quality of life of patients (17). Not only that, but compared to patients without OAB, the cost of medical care for patients with OAB in the U.S. population is going to be more than 2.5 times higher, placing a tremendous financial strain on patients and society (18). However, the specific pathogenesis for OAB are currently unclear. It has been suggested that OAB may be related to obesity, alcohol consumption, diabetes, and lifestyle (19).

However, no studies have focused on the correlation and dose-response relationship between exposure to VOCs and OAB risk. Therefore, to address this gap, we utilized data from the National Health and Nutrition Examination Survey (NHANES) from 2007 to 2020 to investigate the potential association. In this cross-sectional study, we aimed to explore the relationship between exposure to specific VOCs in blood and the risk of developing OAB, which could specifically reflect the level of exposure to specific VOCs in the general population. We conducted a comprehensive retrospective analysis of data on U.S. adults in NHANES through various analytic methods, adjusting for several confounders. This will provide new epidemiologic evidence on the relevance of VOCs exposure to the risk of OAB and may help clinicians to develop more precise prevention and control measures for OAB in the future.

## Participants and methods

### Study population

**Figure 1:**
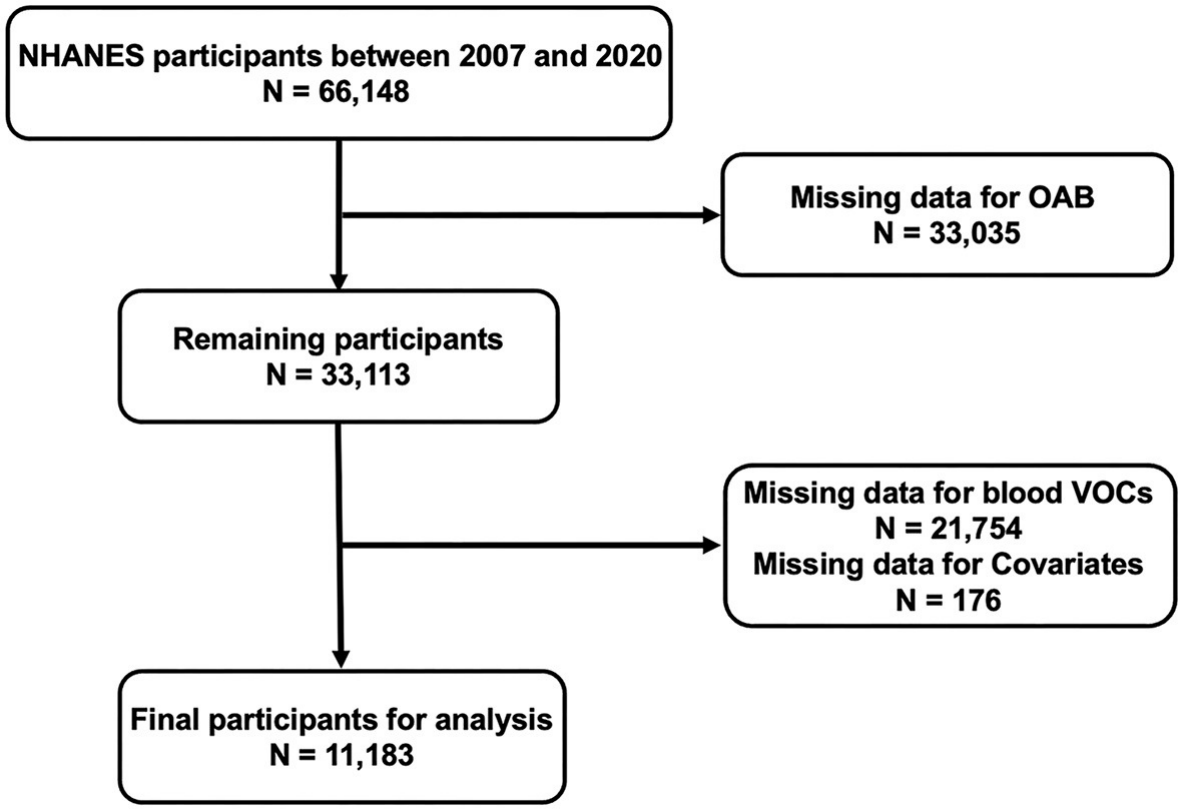
2007–2020 National Health and Nutrition Examination Survey (NHANES) inclusion and exclusion criteria flowchart.

The National Health and Nutrition Examination Survey (NHANES), a national study conducted by the National Center for Disease Control (CDC) and the National Center for Health Statistics for Prevention, was designed to assess the health and nutritional status of adults and children in the United States. The NHANES research protocol was reviewed and approved by the National Center for Health Statistics Research Ethics Review Board. All participants were well-informed and signed an informed consent form. All data are publicly available on the NHANES website (https://www.cdc.gov/nchs/nhanes/). In order to improve statistical accuracy and obtain reliable data, a total of six survey cycles, from 2007 to March 2020, were included in the analysis of our study. Initially, a total of 66,148 participants were included in the six cycles of the survey cycle. Subsequently, we excluded participants without complete information on OAB (*n* =33,035), blood VOCs (*n* = 21,754), and covariates (*n* = 176). Ultimately, 11,183 participants included and analyzed in the present study (Figure 1).

### Measurements of blood VOCs

Volatile organic compounds (VOCs), a large group of chemicals, are widely used in industry and consumer products as solvents, degreasers, and cleaners. Useful information on VOCs exposure and internal dose can be obtained through blood VOCs biomonitoring. Exposure to VOCs was measured primarily through quantification of human blood VOCs and household tap water VOCs. For this purpose, exposure levels to various volatile chemicals were assessed by collecting whole blood samples and analyzing them using stratospheric solid-phase microextraction (SPME)/gas chromatography/isotope dilution mass spectrometry (GC/IDMS). The main advantages of this method include effective detection of VOCs in trace blood samples, low detection thresholds, and suitability for use in a wide range of populations. At least three quality assessment samples were analyzed in each run for quality control purposes. Not only that, but to ensure the quality of the results, sources of contamination were minimized, laboratory efficiency was maintained, and 2% of all samples were rechecked periodically. A total of nine of the most common VOCs exposures studied were included in this study, including blood tetrachloroethene, blood benzene, blood 1,4-dichlorobenzene, blood ethylbenzene, blood o-xylene, blood m-/p-xylene, blood 2,5-dimethylfuran, blood chloroform and blood furan.

### The definition of overactive bladder symptoms

As defined by the International Continence Society, OAB as a symptomatic syndrome characterized by urinary urgency in the absence of urinary tract infection or other obvious pathology usually occurs in conjunction with urinary frequency and nocturia, with or without urge urinary incontinence (UUI) (20). Therefore, OAB should be considered when a patient presents with UUI and nocturia. The following questions were used to define UUI and nocturia: “During the past 12 months, have you leaked or lost control of even a small amount of urine with an urge or pressure to urinate and you couldn't get to the toilet fast enough?” For those who answered “yes,” answer the following question: “Does this happen often?”. Finally, by answering the question, “During the past 30 days, how many times per night did you most typically get up to urinate, from the time you went to bed at night until the time you got up in the morning?” to assess the severity of nocturia. As used in previous studies, the quantification of OAB was assessed by the modified shortened Overactive Bladder Symptom Score (OABSS) questionnaire (19). As shown in Supplementary Table S1, UUI and nocturia symptoms were scored on corresponding scales, and we summed the UUI and nocturia scores to obtain the final OABSS score for each participant. We defined participants with a total score ≥ 3 as overactive bladder patients, the remaining participants with a total score of < 3 and complete questionnaire data were defined as non-overactive bladder patients (19).

### Covariates

In view of previous studies, we included covariates that were considered potential confounders of OAB, including basic demographic information such as age, gender, race, education, and marital status. In addition, a number of lifestyle and health status covariates associated with OAB risk such as body mass index (BMI), history of alcohol consumption, diabetes, hypertension, blood cotinine, blood urea nitrogen, serum creatinine, and serum uric acid were included. Different categorization criteria were used for continuous variables such as body mass index and age when stratifying covariates. The older age group was considered to be ≥50 years of age, while the rest were in the lower age group. Participants were divided into two groups based on BMI: low-normal weight (< 25.0 kg/m^2^) and overweight-obese (≥25.0 kg/m^2^).

### Statistical analysis

As in previous studies, the present study was cross-sectional and utilized NHANES adult data from 2007 to 2020, which contained basic demographic data, laboratory data, and questionnaire data, to analyze the association between exposure to VOCs in blood and risk of OAB (19). In order to minimize sampling error in this study to improve the precision of the results of this study, we combined data from all consecutive years between 2007 and 2020, which contain the complete OAB data and blood concentrations of VOCs in this study. We used the Shapiro-Wilk test for continuous variables to check whether the variables conformed to a normal distribution. For continuous variables that fit a normal distribution are described by the mean and standard deviation (SD). Whereas, continuous variables that do not conform to a normal distribution are described by the mean (upper and lower quartiles). We used frequencies and percentages to characterize the categorical variables. In assessing the clinical characteristics of all participants, we performed different methods of analysis for different variables. Weighted Student *t*-tests were performed for continuous variables. The chi-square tests were used for the categorical variables. Subsequently, in order to explore possible co-exposure trends and co-toxicity effects among blood VOCs, we performed a Spearman's correlation analysis, and the results are presented as a heat map. In addition, we used three different logistic regression models in order to better assess the independent relationship between blood VOCs and OAB risk. Model 1 was adjusted for age, gender, race and BMI. We then further adjusted for education, marital status, alcohol consumption history, and blood cotinine in Model 2. Finally, Model 3 was adjusted for all risk factors including hypertension, diabetes, blood urea nitrogen, serum uric acid, and serum creatinine. On the basis of the results of multivariate logistic regression modeling analyses, we performed log10 conversions of 2,5-dimethylfuran, benzene, and furan in blood and explored potential dose-response relationships between them and OAB risk using restricted cubic spline (RCS) analysis. In addition, we performed subgroup analyses of the three blood VOCs according to age, sex, history of hypertension, and history of alcohol consumption to explore the relationship between different subgroups of the population and the risk of OAB.

Finally, we assessed the relationship between co-exposure to mixed blood VOCs and OAB risk through weighted quantile sum (WQS) regression model after adjusting for all covariates. The WQS is a novel regression model that estimates the overall impact of environmental chemical mixtures on disease-related health outcomes for specific diseases through empirically weighted indices for specific chemicals, which can help identify potentially toxic substances (9, 21). In this study, WQS regression was used to assess the overall co-exposure correlations and the contribution weights of each blood VOCs component for the effect of the nine blood VOCs on OAB risk. The parameters of the WQS regression were set as described in previous studies, 40% for the training set and 60% for the validation set (22). In order to balance the prediction accuracy and generalization ability, we performed 1,000 bootstrap iterations on the training set, which led to the calculation of the WQS index and the weights of each blood VOCs.

All date were analyzed by R (version 4.2.3) and SPSS software (version 26.0). All statistical analyses were performed with *P* < 0.05 as statistically significant differences.

## Results

### Basic characteristics of participants

Table 1 presented the basic characteristics of OAB group and non-OAB group from the 2007–2020 NHANES dataset. In total, 11,183 participants were involved in the study, including 8,727 (78.0%) non-OAB participants and 2,456 (22.0%) OABs participants. The mean age of the participants was 50.03 years and the mean of BMI was 29.45 kg/m^2^. There were significant differences between the non-OAB and OAB groups in age, gender, race, education, marital status, BMI, diabetes, hypertension, alcohol drinking history, blood urea nitrogen, serum creatinine levels and serum uric acid (all *P* < 0.05). Nevertheless, serum uric acid levels were not statistically different between the two groups (*P* > 0.05). More importantly, the distribution of blood VOCs such as blood 2,5 dimethylfuran, blood benzene, blood 1,4-dichlorobenzene and blood furan were statistically significant between the non-OAB group and the OAB group, which may indicate that there were differences in exposures between the two groups (all *P* < 0.05). However, the remaining exposure to VOCs was not significantly different between the two groups.

**Table 1:** Baseline characteristics of NHANES participants between 2007 and 2020 (*n* = 11,183)^a^.

**Characteristic**	**All**	**Non-overactive bladder**	**Overactive bladder**	***P*-value**
	**Patients**	**No. (%)**	**No. (%)**	
	***N*** = **11,183**	***N*** = **8,727 (78.0)**	***N*** = **2,456 (22.0)**	
Age				< 0.001
Mean (IQR)	50.03 [35.00, 64.00]	46.00 [33.00, 61.00]	62.00 [50.00, 72.00]	< 0.001
< 50 years	8,727 (78.0)	4,855 (89.0)	599 (11.0)	
≥50 years	2,456 (22.0)	3,872 (44.4)	1,857 (75.6)	
Gender				< 0.001
Male	5,553 (49.7)	4,523 (51.8)	1,030 (41.9)	
Female	5,630 (50.3)	4,204 (48.2)	1,426 (58.1)	
Race				< 0.001
Mexican American	1,679 (15.0)	1,331 (15.3)	348 (14.2)	
Other Hispanic	1,201 (10.7)	926 (10.6)	275 (11.2)	
Non-Hispanic white	4,442 (39.7)	3,527 (40.4)	915 (37.3)	
Non-Hispanic black	2,412 (21.6)	1,703 (19.5)	709 (28.9)	
Other race	1,449 (13.0)	1,240 (14.2)	209 (8.5)	
Education				< 0.001
≤ High school	5,060 (45.2)	3,697 (73.1)	1,363 (26.9)	
>High school	6,123 (54.8)	5,030 (57.6)	1,093 (44.5)	
Marital status				< 0.001
Married	5,991 (53.6)	4,793 (54.9)	1,198 (48.8)	
Unmarried and others	5,192 (46.4)	3,934 (75.8)	1,258 (24.2)	
BMI (kg/m^2^)				< 0.001
Mean (IQR)	29.45 [24.60, 32.90]	27.80 [24.30, 32.20]	30.20 [25.87, 35.10]	
< 25.0	3,045 (27.2)	2,560 (84.1)	485 (15.9)	
≥25.0	8,138 (72.8)	6,167 (70.7)	1,971 (80.3)	
Hypertension				< 0.001
Yes	4,164 (37.2)	2,769 (31.7)	1,395 (56.8)	
No	7,019 (62.8)	5,958 (84.9)	1,061 (15.1)	
Diabetes				< 0.001
Yes	1,514 (13.5)	901 (10.3)	613 (25.0)	
No	9,669 (86.5)	7,826 (80.9)	1,843 (19.1)	
Alcohol drinking history				< 0.001
Yes	8,654 (77.4)	6,873 (78.8)	1,781 (72.5)	
No	2,529 (22.6)	1,854 (73.3)	675 (26.7)	
Blood cotinine (ng/mL)	54.92 (126.23)	54.82 (126.84)	55.29 (124.08)	0.870
Blood urea nitrogen (mg/dL)	14.11 [10.00, 17.00]	13.00 [10.00, 16.00]	14.00 [11.00, 18.00]	< 0.001
Serum creatinine (mg/dL)	0.85 [0.71, 1.01]	0.84 [0.71, 1.00]	0.86 [0.72, 1.05]	< 0.001
Serum uric acid (mg/dL)	5.30 [4.40, 6.40]	5.30 [4.40, 6.30]	5.40 [4.50, 6.60]	< 0.001
Blood VOCs (ng/mL)				
Blood 2,5-Dimethylfuran	0.01 [0.01, 0.01]	0.01 [0.01, 0.01]	0.01 [0.01, 0.01]	0.001
Blood tetrachloroethene	0.03 [0.03, 0.03]	0.03 [0.03, 0.03]	0.03 [0.03, 0.03]	0.079
Blood benzene	0.06 (0.14)	0.06 (0.13)	0.07 (0.14)	0.027
Blood 1,4-dichlorobenzene	0.04 [0.03, 0.16]	0.03 [0.03, 0.15]	0.05 [0.03, 0.19]	< 0.001
Blood ethylbenzene	0.07 (1.35)	0.07 (1.49)	0.06 (0.57)	0.668
Blood o-xylene	0.06 (1.19)	0.06 (1.32)	0.05 (0.46)	0.605
Blood m-/p-xylene	0.22 (6.34)	0.24 (7.08)	0.17 (2.16)	0.660
Blood furan	0.02 [0.02, 0.02]	0.02 [0.02, 0.02]	0.02 [0.02, 0.02]	< 0.001
Blood chloroform	0.02 (0.13)	0.01 (0.03)	0.02 (0.26)	0.132

NHANES, National Health and Nutrition Examination Survey; VOCs, volatile organic compounds; SD, standard deviation; IQR, interquartile range; BMI, body mass index.

^a^For categorical variables, *P*-values were analyzed by chi-square tests. For continuous variables, *P*-values were derived from Student's *t*-test.

### Spearman correlation analysis among blood VOCs

**Figure 2:**
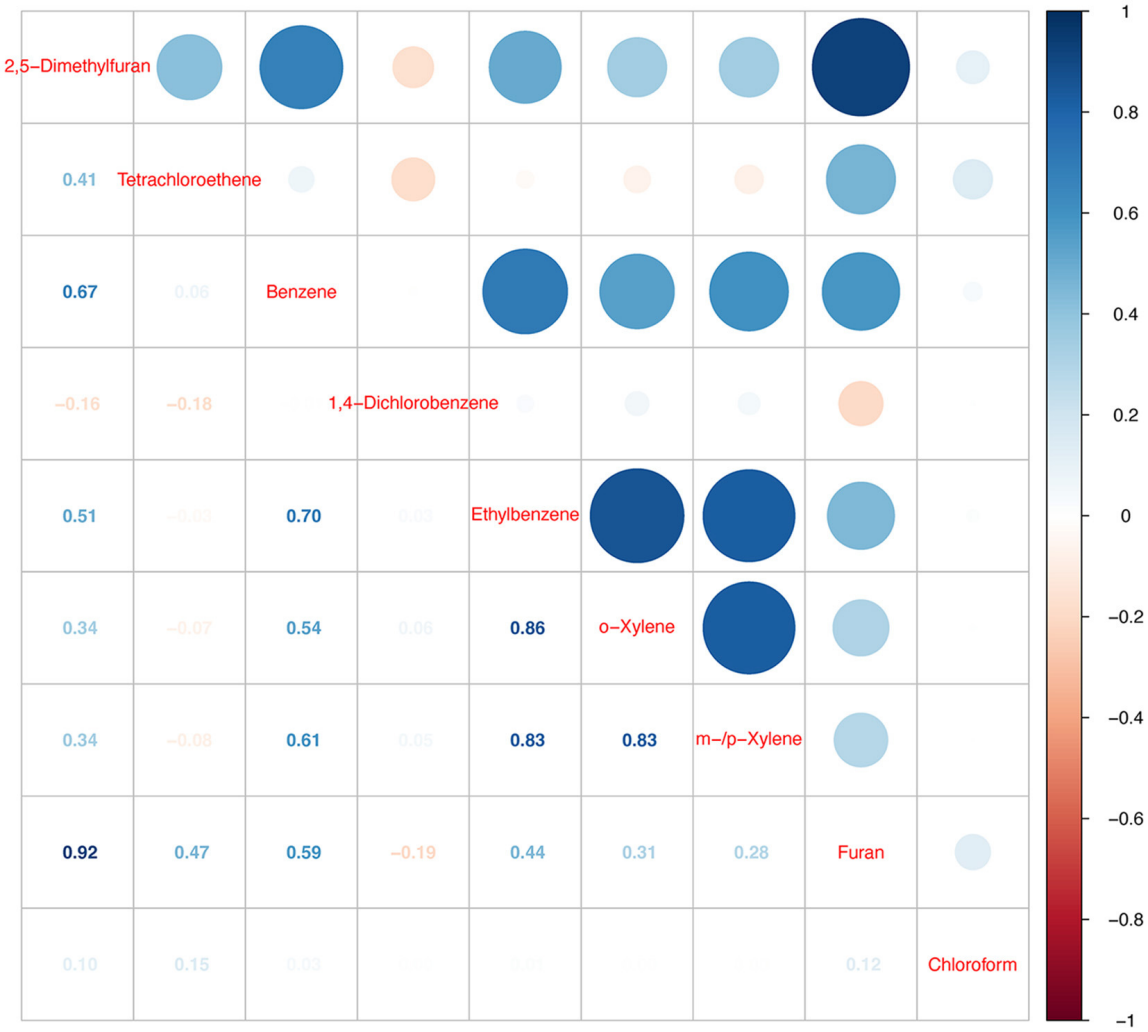
Heatmap of Spearman's correlation model analysis.

To investigate potential co-exposure patterns of the nine blood VOCs, we performed a Spearman correlation modeling analysis (Figure 2). More specifically, blood 2,5-dimethylfuran was highly positively correlated with blood furans (*r* = 0.92). Moreover, we found a significant correlation between blood m-/p-xylene and blood o-xylene (*r* = 0.83) and ethylbenzene (*r* = 0.83), whereas blood ethylbenzene had a strong co-exposure with blood o-xylene (*r* = 0.86).

### Associations between blood VOCs and OAB

Multivariate logistic regression analyses were used to investigate the association between exposure to blood VOCs and OAB risk after adjusting for the potential confounders included (Table 2). The results indicated that in model 3, with each unit increase in the concentration of 2,5-dimethylfuran (aOR = 2.940, 95% CI: 1.096–7.890, *P* = 0.032), benzene (aOR = 1.460, 95% CI: 1.044–2.043, *P* = 0.027) and furan (aOR = 9.426, 95% CI: 1.421–62.500, *P* = 0.020) in the blood, there was a corresponding 194.0, 46.0, and 842.5% increase in the risk of developing OAB, respectively. The above results suggest that long-term exposure to environmental 2,5-dimethylfuran, benzene and furan may be an independent risk factor for OAB.

**Table 2:** Association [adjusted odds ratio (95% confidence intervals)] between OAB and blood VOCs levels in NHANES participants between 2007 and 2020^a^.

**VOCs exposure**	**Model 1**		**Model 2**		**Model 3**	
	**aOR (95% CI)**	***P*** **-value**	**aOR (95% CI)**	***P*** **-value**	**aOR (95% CI)**	***P*** **-value**
Blood 2,5-dimethylfuran	3.272 (1.563–6.848)	0.002	2.762 (1.030–7.405)	0.043	2.940 (1.096–7.890)	0.032
Blood tetrachloroethene	0.989 (0.883–1.108)	0.849	0.981 (0.873–1.103)	0.752	0.984 (0.877–1.103)	0.779
Blood benzene	1.829 (1.291–2.592)	0.001	1.639 (1.082–2.483)	0.020	1.460 (1.044–2.043)	0.027
Blood 1,4-dichlorobenzene	1.003 (0.996–1.010)	0.430	1.003 (0.996–1.010)	0.681	1.001 (0.994–1.008)	0.727
Blood ethylbenzene	0.997 (0.948–1.050)	0.922	0.996 (0.942–1.052)	0.882	0.999 (0.950–1.051)	0.976
Blood o-xylene	0.992 (0.924–1.066)	0.827	0.991 (0.919–1.069)	0.811	0.995 (0.930–1.065)	0.884
Blood m-/p-xylene	0.999 (0.988–1.011)	0.916	0.999 (0.988–1.011)	0.905	1.000 (0.989–1.011)	0.984
Blood furan	10.843 (2.584–45.498)	0.001	8.135 (1.236–53.534)	0.029	9.425 (1.421–62.500)	0.020
Blood chloroform	1.220 (0.831–1.790)	0.310	1.195 (0.815–1.752)	0.361	1.198 (0.817–1.756)	0.354

NHANES, National Health and Nutrition Examination Survey; VOCs, volatile organic compounds; CI, confidence interval; aOR, adjusted odds ratio; OAB, overactive bladder.

^a^Model 1: adjusted basic information for age, gender, race and BMI.

Model 2: model 1 further adjusted lifestyle factors for education, marital status, alcohol drinking history and blood cotinine.

Model 3: model 2 fully adjusted for hypertension, diabetes, blood urea nitrogen, serum uric acid and serum creatinine.

**Figure 3:**
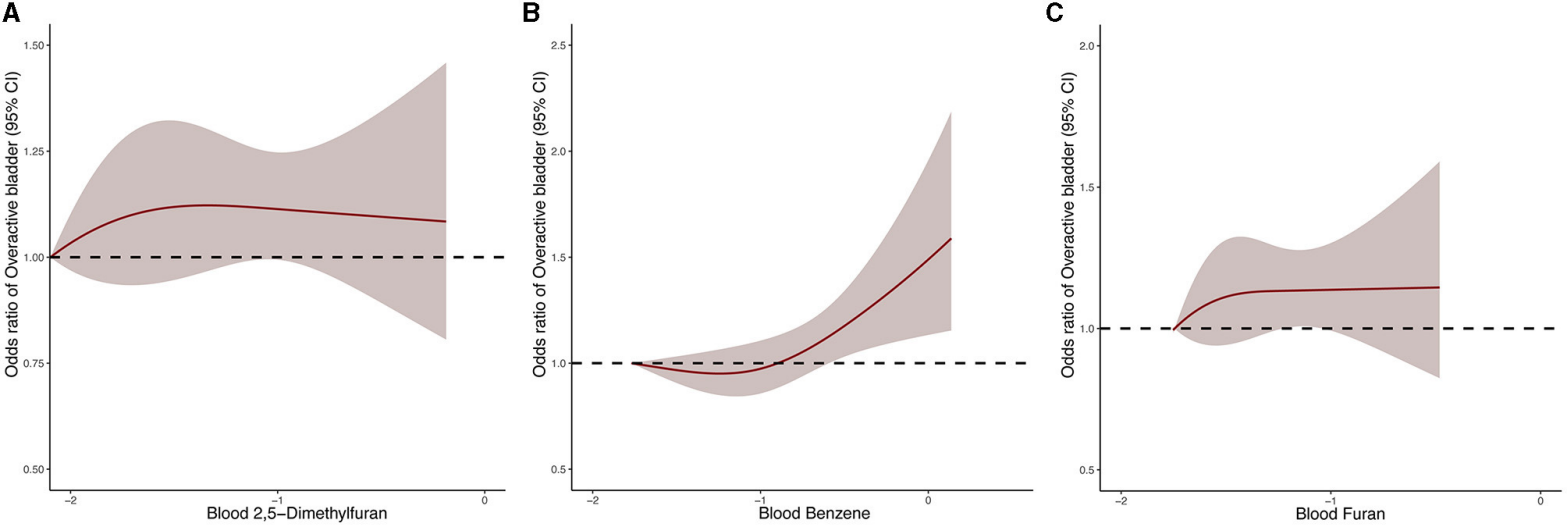
Dose–response relationship between blood 2,5-dimethylfuran **(A)**, blood benzene **(B)**, blood furan **(C)** and the risk of OAB.

In order to further investigate the dose-response relationship between blood VOCs and OAB risk, we performed a fully adjusted restricted cubic spline analysis of 2,5-dimethylfuran, benzene and furan in blood (Figure 3). The results showed that the risk of OAB increased significantly with increasing concentrations of the three blood VOCs after adjusting for all potential risk factors. However, the OAB risk associated with 2,5-dimethylfuran and furan increased dramatically in the early part of the exposure period and the curve stabilized in the late part of the exposure period.

### WQS regression analysis of associations between blood VOCs and OAB

**Figure 4:**
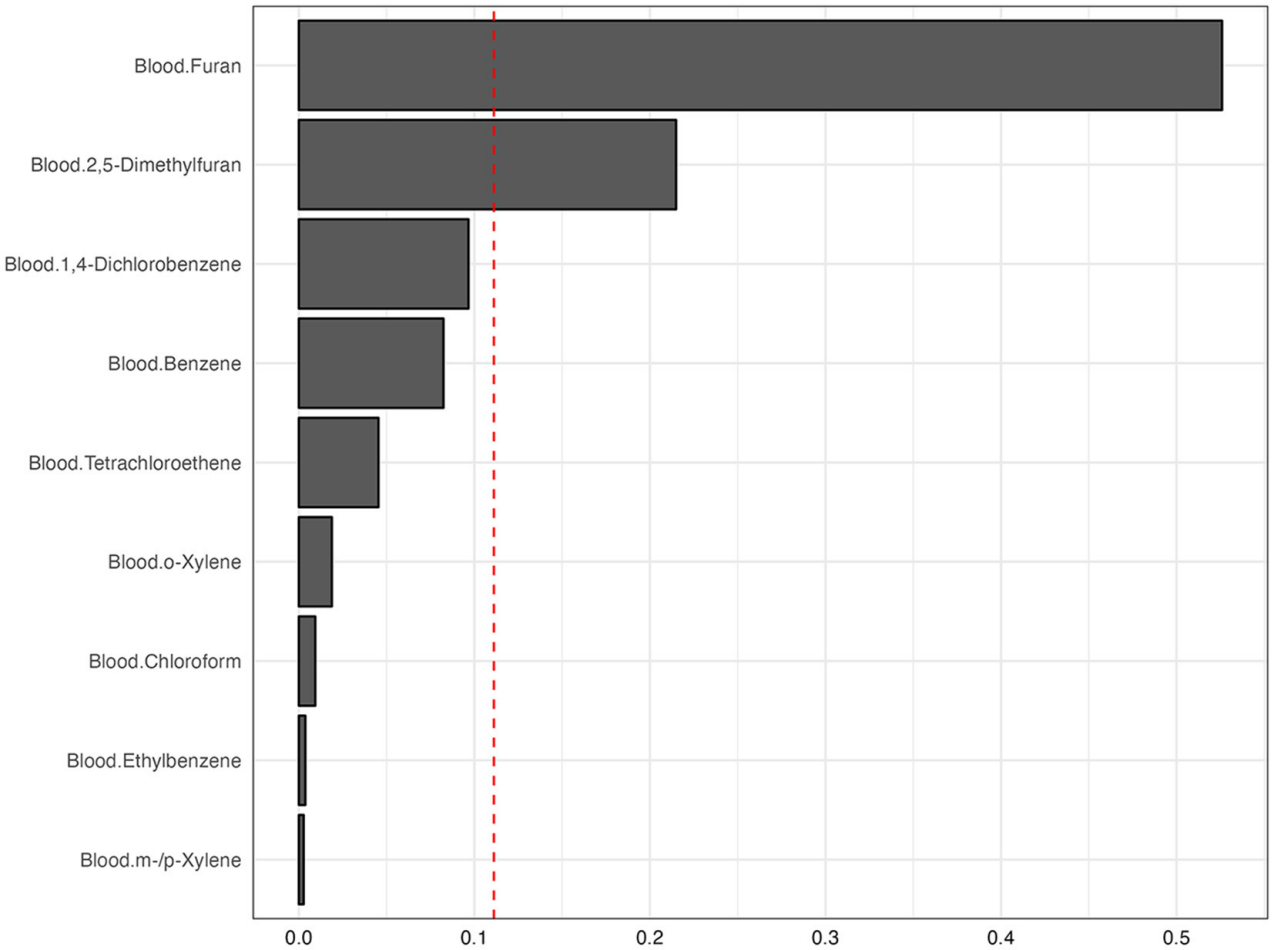
The WQS regression model estimated the weights of each blood VOCs associated with OAB.

We used WQS regression models to assess the potential association between co-exposure to blood VOCs and OAB risk (Figure 4). Significant positive associations between the WQS index and the risk of OAB after adjusting for all covariates were demonstrated. After adjustment, the risk of OAB increased by 29% (OR = 1.29, 95% CI: 1.11–1.49) for each unit increase in WQS index, and furans were the most important component of all blood VOCs, with a contribution weight of 52.59%.

### Subgroup analysis

We performed subgroup analyses based on age, gender, hypertension, and alcohol drinking history to investigate stratified associations between blood VOCs and OAB risk in different subgroups (Table 3). The results showed a significant correlation between 2,5-dimethylfuran, benzene and furan in blood and OAB risk in specific subgroups. Specifically, for participants under 50 years of age, increased blood concentrations of VOCs significantly enhanced the risk of OAB, with furans having the greatest risk (aOR = 54.733, 95% CI: 6.113–490.087, *P* < 0.001). In the subgroup of male, blood 2,5-dimethylfuran (aOR = 5.416, 95% CI: 1.558–18.834, *P* = 0.008), blood benzene (aOR = 1.751, 95% CI: 1.042–2.945, *P* = 0.035) and blood furan (aOR = 24.656, 95% CI: 2.204–300.342, *P* = 0.012) concentrations were positively associated with the risk of OAB. In addition, this positive association between the 3 blood VOCs and the risk of OAB was also observed in the subgroups of non-hypertensive and alcohol drinkers.

**Table 3:** Subgroup analysis between OAB and blood VOCs levels in NHANES participants between 2007 and 2020^a^.

**Subgroups**	**Model 3**	
	**aOR (95% CI)**	***P*** **-value**
**Age (years)**		
<**50**		
Blood 2,5-dimethylfuran	5.814 (1.996–16.940)	0.001
Blood benzene	3.015 (1.721–5.280)	< 0.001
Blood furan	54.733 (6.113–490.087)	< 0.001
≥**50**		
Blood 2,5-dimethylfuran	1.752 (0.416–7.376)	0.445
Blood benzene	1.349 (0.848–2.147)	0.206
Blood furan	3.236 (0.247–42.367)	0.371
**Gender**		
**Male**		
Blood 2,5-dimethylfuran	5.416 (1.558–18.834)	0.008
Blood benzene	1.751 (1.042–2.945)	0.035
Blood furan	24.656 (2.204–300.342)	0.012
**Female**		
Blood 2,5-dimethylfuran	2.576 (0.120–55.414)	0.546
Blood benzene	1.492 (0.727–3.063)	0.276
Blood furan	0.984 (0.176–5.507)	0.985
**Hypertension**		
**Yes**		
Blood 2,5-dimethylfuran	1.630 (0.377–7.039)	0.513
Blood benzene	1.837 (1.009–3.344)	0.046
Blood furan	3.309 (0.194–47.542)	0.428
**No**		
Blood 2,5-dimethylfuran	4.086 (1.487–11.229)	0.006
Blood benzene	1.735 (1.069–2.816)	0.026
Blood furan	17.953 (2.475–130.242)	0.004
**Alcohol drinking history**		
**Yes**		
Blood 2,5-dimethylfuran	2.285 (1.021–5.113)	0.044
Blood benzene	1.596 (1.116–2.282)	0.010
Blood furan	6.490 (1.354–31.116)	0.019
**No**		
Blood 2,5-dimethylfuran	0.744 (0.017–33.559)	0.879
Blood benzene	0.879 (0.158–4.892)	0.883
Blood furan	2.350 (0.714–7.736)	0.686

NHANES, National Health and Nutrition Examination Survey; OAB, overactive bladder; VOCs, volatile organic compounds; CI, confidence interval; aOR, adjusted odds ratio.

^a^Model 3: adjusted all factors for age, gender, race, BMI, marital status, education, hypertension, hypertension, diabetes, alcohol drinking history, blood urea nitrogen, blood cotinine, serum uric acid and serum creatinine.

## Discussion

In the present study, we conducted a nationally representative cross-sectional study to systematically investigate the potential relationship between the risk of OAB and exposure to specific VOCs in U.S. adults. To our knowledge, this is the first time to investigate the association between blood VOCs and the risk of OAB with nationally representative data. Our results suggested a significant relationship between blood concentrations of VOCs and the risk of OAB, even though a variety of potential confounders were considered. Specifically, we found that blood concentrations of 2,5-dimethylfuran, benzene and furan significantly and positively associated with OAB risk by performing multivariate logistic regression model analysis and restricted cubic spline analysis. In addition, the WQS model analysis was used to assess the co-exposure effects of blood VOCs mixtures on OAB risk. After adjusting for potential confounders, furans in blood had the highest weights. Finally, in subgroup analyses, younger, male, non- hypertension and alcohol drinking adults were more likely to be affected by elevated blood 2,5-dimethylfuran, benzene and furan concentrations thereby increasing the risk of OAB compared to older, female, hypertensive and non-drinking adults.

As a group of urologic disorders that severely affect patients' lives and spirituality, the underlying mechanisms of OAB are unknown. Some lifestyles including diet and water intake have a significant impact on OAB (23, 24). Dallosso et al. investigated the role of diet and lifestyle in OAB by studying 7,046 women over the age of 40. A significant relationship was found between OAB symptoms and obesity, smoking and carbonated beverage consumption. Eating more vegetables, bread and chicken reduced the risk of developing OAB symptoms (25). In a retrospective intervention study, it was found that fluid intake needs to be individualized and carefully tested for patient status, and that increasing fluid intake did not improve OAB symptoms (24). Moreover, previous studies had suggested that OAB may be associated with a variety of factors, including metabolic syndrome, affective disorders, and sex hormone disorders (26). While the effects of OAB may contribute to the susceptibility of affected individuals to anxiety and depression, several studies indicate that emotional stress and anxiety or depression could be risk factors for the progression and development of OAB in women (27). It had been shown that serotonin depletion was considered to be a common pathophysiologic candidate for anxiety or depression and OAB, and that decreased serotonin levels in the central nervous system were accompanied by urinary frequency and detrusor overactivity (28, 29). Not only that, but central sensitization, the enhanced response of injured neurons within the central nervous system to normal or subthreshold afferent neurons, has recently been recognized as a common pathophysiological factor in anxiety or depression and OAB (30). In addition, some cerebrovascular diseases such as stroke can lead to OAB. After the acute phase of stroke, OAB is the main lower urinary tract symptom in post-stroke patients (31). Akkoç et al. found that post-stroke patients experienced urinary urgency in about two-thirds of patients at 6-month follow-up (32). In a recent study of U.S. adults, a positive correlation was found between exposure to VOCs and cardio-cerebrovascular events, including benzene, m-/p-xylene, o-xylene, and ethylbenzene (33). In addition, a study of 2011–2020 National Health Survey data found that exposure to VOCs may increase the risk of depression. VOCs, including acrylonitrile, toluene, styrene, acrylamide, 1,3-butadiene, and xylene, may be associated with an increased risk of depression through damage to the nervous system (9). Excessive exposure to benzene compounds, such as toluene, xylene and styrene, can lead to neurocognitive impairment and central nervous system disorders (34, 35). Given previous studies, exposure to VOCs may affect affective psychiatric disorders as well as cerebrovascular disease, leading to an increased risk of OAB.

Previous epidemiologic studies had found that sex hormone disorders were an important cause of lower urinary tract symptoms because of the display of estrogen and progesterone receptors in the urinary tract, bladder, and pelvic floor muscles. Up to 70% of women with urinary incontinence begin after their last menstrual period (36). In a randomized controlled trial, Nelken et al. found that the use of estradiol-releasing vaginal ring in postmenopausal patients with OAB was able to reduce the number of times a patient urinated per day. This further confirmed the role of hormones in the development of OAB (37). In addition, sex hormone disorders were equally causative of OAB in male patients. It was found that testosterone may decrease excitability of the urethral muscle, ameliorate bladder wall fibrosis, and may affect the release of uroepithelial mediators, providing a theoretical basis for the pathogenic role of androgen deficiency in the pathogenesis of OAB (38–41). Wei et al. found in a cross-sectional study that exposure to 2,5-dimethylfuran and benzene may lead to endocrine abnormalities associated with female sex hormones, particularly in underweight and normal weight women (42). Not only that, but there was a significant association between VOC exposure and increased testosterone, estradiol, and sex hormone-binding globulins (43). According to the studies mentioned above, sex hormone disorders lead to an increased risk of developing OAB. Therefore, it is interesting to explore whether exposure to VOCs may contribute to the development of OAB by interfering with sex hormone metabolism.

Although the specific mechanisms by which VOCs are involved in OAB are not fully understood, potential biological mechanisms between VOCs and OAB may involve oxidative stress and chronic inflammation induced by VOCs exposure. Previous studies have reported an increase in the amount of pro-inflammatory mediators in the bladder and urine of patients with OAB (44–46). Excessive oxidative stress plays a key role in the development of OAB (47, 48). Oxygen and nutrients supplied in the blood provide for the realization of normal storage and voiding functions of the bladder. When there is a decrease in blood saturation, hypoxia is induced, accompanied by a high level of oxidative free radicals. Thus, the periodic accumulation of oxidative stress in the bladder leads to the development of OAB (48, 49). And long-term exposure to VOCs has been shown to be involved in oxidative stress and chronic inflammatory processes (50, 51). VOCs promote oxidative stress in the vivo, and high levels of oxidative stress stimulate and activate the production of inflammatory mediators. As inflammatory mediators continue to increase, systemic chronic inflammatory stress will continue to develop (52, 53). A large-scale adjustment from China revealed that daily exposure to environmental VOCs triggers oxidative damage (54). However, the specific molecular biological mechanisms of how VOCs contribute to the increased risk of OAB need to be confirmed by further exploration in the future.

Nevertheless, it should be noted that this study has several limitations. First, this study was a cross-sectional study and could not confirm the causal relationship and pathologic mechanisms between VOCs and OAB. Second, we could not avoid other confounders that were not included and measured, such as diet and water intake, although we adjusted for the various confounders associated with OAB risk to the extent possible. Finally, because this study was based on a study conducted among U.S. adults, the results need to be further investigated in other countries and populations. This study provided a novel scientific basis for exploring the relationship between exposure to VOCs and OAB risk. Prospective studies with large sample sizes and multiple regions are needed in the future to explore the causal and pathological mechanisms between VOCs exposure and OAB.

## Conclusion

Our study provides novel evidence for an positively and independently association between specific blood VOC exposures and the risk of developing OAB in U.S. adults, with blood furan being the most significant. The results indicated that long-term exposure to VOCs may lead to an increased risk of developing OAB in adults, especially blood 2,5-dimethylfuran, benzene and furan. In addition, the concentration association between blood 2,5-dimethylfuran, benzene and furan and OAB risk suggests that long-term exposure to VOCs may lead to an increased risk of OAB, especially in the initial period presenting a significant increase. The risk of OAB occurrence is more likely to be influenced by blood VOCs in certain specific populations, including young and middle-aged, male, non-hypertensive, and alcohol-drinking populations. Finally, more prospective and experimental studies are needed to further validate the conclusions of this study and explore the pathological mechanisms.

## Data availability statement

The original contributions presented in the study are included in the article/Supplementary material, further inquiries can be directed to the corresponding authors.

## Ethics statement

This study used previously collected de-identified data, which had been reviewed and approved by the National Center for Health Statistics (NCHS) Research Ethics Review Committee. The studies were conducted in accordance with the local legislation and institutional requirements. The participants provided their written informed consent to participate in this study.

## Author contributions

DZ: Conceptualization, Data curation, Formal analysis, Investigation, Methodology, Project administration, Resources, Software, Supervision, Validation, Visualization, Writing – original draft, Writing – review & editing. ZY: Formal analysis, Project administration, Resources, Validation, Visualization, Writing – original draft, Writing – review & editing. JH: Conceptualization, Data curation, Investigation, Methodology, Software, Supervision, Writing – original draft, Writing – review & editing. YY: Conceptualization, Data curation, Investigation, Methodology, Software, Supervision, Validation, Visualization, Writing – original draft, Writing – review & editing. KL: Conceptualization, Data curation, Formal analysis, Funding acquisition, Investigation, Methodology, Project administration, Resources, Software, Supervision, Validation, Visualization, Writing – original draft, Writing – review & editing.
