# Supplementary material for: The exposure to volatile organic compounds associate positively with overactive bladder risk in U.S. adults: a cross-sectional study of 2007–2020 NHANES
Source: Front Public Health. 2024 Jun 7;12:1374959. doi: 10.3389/fpubh.2024.1374959 (PMC11190323; doi:10.3389/fpubh.2024.1374959)
Supplement: Supplementary file 1 [file Data_Sheet_1.docx]

**Table S1:** Criteria for conversion of symptom frequencies recorded in NHANES to

OABSS scores.^a^

| **Urge urinary incontinence frequency**  **(according to NHANES)** | **Urge urinary incontinence score**  **(according to OABSS)** |
| --- | --- |
| Every day and/or night | 3 |
| A few times a week | 2 |
| A few times a month | 1 |
| Less than once a month | 1 |
| Never | 0 |
| **Nocturia frequency(from NHANES)** | **Nocturia score (according to OABSS)** |
| 5 or more | 3 |
| 4 | 3 |
| 3 | 3 |
| 2 | 2 |
| 1 | 1 |
| 0 | 0 |

**Abbreviations:**

^a^ NHANES, National Health and Nutrition Examination Survey; OABSS, Overactive Bladder Symptom Score.
